# Persistent treatment with cholinesterase inhibitors and/or memantine slows clinical progression of Alzheimer disease

**DOI:** 10.1186/alzrt7

**Published:** 2009-10-21

**Authors:** Susan D Rountree, Wenyaw Chan, Valory N Pavlik, Eveleen J Darby, Samina Siddiqui, Rachelle S Doody

**Affiliations:** 1 Department of Neurology, Baylor College of Medicine, 6550 Fannin, Suite 1801, Houston, TX 77030, USA; 2 Division of Biostatistics, University of Texas School of Public Health, Division of Biostatistics, 1200 Herman Pressler, Suite 846, Houston, TX 77030, USA; 3 Department of Community and Family Medicine, Baylor College of Medicine, 3701 Kirby Drive, Houston, TX 77098, USA; 4 Department of Psychiatry, University of Texas Health Science Center, 2800 S MacGregor Way, Houston, TX 77021, USA

## Abstract

**Introduction:**

There are no empiric data to support guidelines for duration of therapy with antidementia drugs. This study examined whether persistent use of antidementia drugs slows clinical progression of Alzheimer disease (AD) assessed by repeated measures on serial tests of cognition and function.

**Methods:**

Six hundred forty-one probable AD patients were followed prospectively at an academic center over 20 years. Cumulative drug exposure was expressed as a persistency index (PI) reflecting total years of drug use divided by total years of disease symptoms. Baseline and annual testing consisted of Mini-Mental State Examination (MMSE), Alzheimer's Disease Assessment Scale-Cognitive Subscale (ADAS-Cog), Baylor Profound Mental Status Examination (BPMSE), Clinical Dementia Rating-Sum of Boxes (CDR-SB), Physical Self-Maintenance Scale (PSMS), and Instrumental Activities of Daily Living (IADL). Annual change in slope of neuropsychological and functional tests as predicted by follow-up time, PI, and the interaction of these two variables was evaluated.

**Results:**

PI was associated with significantly slower rates of decline (with, without adjustment for covariates) on MMSE (*P *< 0.0001), PSMS (*P *< 0.05), IADL (*P *< 0.0001), and CDR-SB (*P *< 0.001). There was an insignificant trend (*P *= 0.053) for the PI to be associated with slower rate of decline on BPMSE. The association of PI with ADAS-Cog followed a quadratic trend (*P *< 0.01). Analysis including both linear and quadratic terms suggests that PI slowed ADAS-Cog decline temporarily. The magnitude of the favorable effect of a rate change in PI was: MMSE 1 point per year, PSMS 0.4 points per year, IADL 1.4 points per year, and CDR-SB 0.6 points per year. The change in mean test scores is additive over the follow-up period (3 ± 1.94 years).

**Conclusions:**

Persistent drug treatment had a positive impact on AD progression assessed by multiple cognitive, functional, and global outcome measures. The magnitude of the treatment effect was clinically significant. Positive treatment effects were even found in those with advanced disease.

## Introduction

Since 1993, five drugs have been marketed for the treatment of Alzheimer disease (AD). These treatments are sometimes regarded as having only 'symptomatic' rather than 'disease-modifying' effects, although the utility of this distinction has been questioned [1]. The first cholinesterase inhibitor (ChEI) approved specifically to treat symptoms of AD was tacrine, but it is no longer used. Galantamine and rivastigmine (both approved for use in mild to moderate AD) and donepezil (approved for use in mild to severe AD) are reported to benefit cognition, function, and behavior in AD patients [2-9]. Donepezil confers significant benefits in controlled studies lasting up to 1 year [10,11] in mild to moderate AD, and 6-month studies have reported that therapy is efficacious in severe and institutionalized patients [12-15]. One controlled 2-year study (AD2000) evaluated mild to moderate AD patients and found no significant differences between patients who took donepezil compared with placebo in institutionalization rates or progression to disability, but significant cognitive and functional effects were maintained in those who received active treatment, despite methodological flaws [16,17].

Memantine modulates glutamate via *N*-methyl-D-aspartate (NMDA) receptor antagonism and was approved for treatment of moderate to severe AD based upon three pivotal trials. The first was a 12-week study of nursing home residents [18]. In a 7-month study, memantine monotherapy compared with placebo was beneficial [19], and in a 6-month trial, combination therapy with memantine plus donepezil was superior to placebo plus donepezil [20]. A recently published 24-week trial that also evaluated moderate to severe AD patients suggested a weaker treatment effect with memantine monotherapy; treatment was not associated with significant overall cognitive or global changes in this trial, although there were apparent benefits at intermediate time points [21]. Observations from the pivotal trials indicate that early initiation of treatment is associated with greater long-term benefit and that withdrawal and re-initiation of treatment is detrimental [22].

Observational studies suggest that these drugs may benefit cognition for a year [23] or slow time to dementia-related nursing home placement [24,25]. Two recent studies have evaluated the long-term use of a ChEI alone compared with a drug regimen including both a ChEI and memantine. In one study, AD patients followed for an average of 30 months taking a ChEI plus memantine had slower cognitive and functional decline compared with those taking only a ChEI or those receiving no treatment [26]. In another study, AD patients followed for an average of 5 years had significant delay until nursing home admission if they had used a ChEI, and the effect was significantly augmented if they had used a ChEI plus memantine [27]. The ethical dilemma of prolonged exposure to placebo limits longer-duration randomized trials, so only a practice-based population can provide longer observation [28].

No studies have evaluated the optimal duration of therapy or whether greater persistency of antidementia drug therapy affects patient outcomes. We hypothesized that greater cumulative exposure to the ChEIs or memantine or both would be associated with slower rates of decline on cognitive and functional measures in AD patients over the long term. We assessed the impact of persistent treatment from the onset of symptoms on key measures of disease progression. This study was made possible by the longitudinal database at the Baylor College of Medicine (BCM), in which medication exposure throughout the disease course is quantified, serial measurements of cognition and function are collected annually, and the majority of patients are followed from diagnosis to death.

## Materials and methods

### Subjects

All members of this cohort agreed to participate in a database approved by the institutional review board at BCM and met criteria for the diagnosis of probable AD as determined by the National Institute of Neurological and Communicative Disorders and Stroke and the Alzheimer's Disease and Related Disorders Association [29]. All probable AD patients with at least one complete comprehensive annual follow-up evaluation were eligible. All patients underwent an evaluation by a neurologist and completed a standardized dementia workup that has been in continuous use since 1989 [30]. A detailed history and interview with the patient and an informant, neurological and physical examinations, a neuroimaging study of the brain, neuropsychological testing, and screening laboratory studies were performed at the initial visit. The duration of illness is estimated by the physician by a standardized procedure reported to the nearest half-year [31]. We use a standardized method to estimate duration of illness in AD patients. There is a set of questions for generating an estimate regarding the date of first symptoms to the nearest half-year. Physicians revise this estimate in conjunction with medical record review and patient/informant interviews and by testing the estimate by recall of life events. Inter-rater reliability for the estimate has been reported to be 0.95. The initial battery of neuropsychological tests is repeated along with neurological and physical exams annually. Each patient's diagnosis is reviewed at a consensus conference held weekly at BCM following the baseline and annual testing. Vital status was obtained from the National Death Index every 6 months, and the last inquiry was made in March 2006 with a censoring date for the analysis on 31 December 2005. At censoring, 54% of the cohort had died and 46% were alive.

### Persistency index

Persistent drug exposure was calculated by a persistency index (PI): total years of drug use divided by the total years of disease symptoms. This index includes the time period before the new patient visit. The number of years of disease symptoms was determined by the physician's estimate at the initial visit and extended to the last outcome assessment date.

### Exposure

Exposure to the antidementia drugs was ascertained for each subject. Early drug exposure information (prior to the initial visit) was recorded by the physician at the first clinic visit by history obtained from the patient and caregiver along with a review of medical records. The clinician seeing the patient completed a drug exposure form at each clinic visit and recorded all start dates and end dates, if applicable, for use of ChEIs, memantine, and other antidementia drugs. Lapses in treatment or switching from one drug to another was also noted and recorded by the treating physician. The dates of drug exposure were concurrently entered in the electronic database so the cumulative time on medication could be determined. We used chart review to complete drug exposure forms for the subjects who had been seen prior to the implementation of the drug exposure form in 2002. We also reconciled any exposure that occurred by virtue of participation in a clinical research trial by reviewing treatment assignments and study records. Exposure to antidementia drugs was calculated in months of use. No attempt was made to quantify the dose or to distinguish between outcomes on monotherapy with a ChEI or combination therapy with a ChEI and glutamate modulator. Historically, ChEIs were widely available after the introduction of tacrine in 1993, donepezil in 1996, rivastigmine in 2000, and galantamine in 2001. Combination therapy or multiple-drug use did not often occur until memantine was approved in October 2003.

### Neuropsychological testing

Serial measures of cognition pertinent to this analysis included the Mini-Mental State Examination (MMSE) [32], a widely used dementia severity test with scores that range from 0 to 30 points. Higher scores reflect better performance. The Baylor Profound Mental Status Examination (BPMSE) [33] was administered when the MMSE score was 10 or below. The BPMSE is sensitive to longitudinal change and evaluates decline in advanced-stage AD when performance reaches lowest levels on conventional measures. It is modeled after the MMSE and consists of 25 patient-derived cognitive questions that assess orientation, language, attention, and motor functioning. Higher scores reflect better cognitive performance. The Alzheimer's Disease Assessment Scale-Cognitive Subscale (ADAS-Cog) [34] measures cognitive domains often impaired in AD, including memory, language, and praxis. The error score ranges from 0 to 70, and higher scores reflect greater cognitive impairment. The ADAS-Cog is thought to be more sensitive to smaller changes in cognitive function than the MMSE but is less widely used clinically. The global measure was the Clinical Dementia Rating-Sum of Boxes (CDR-SB) [35-37]. The score is derived from a patient interview in conjunction with an interview of a collateral source. The CDR-SB is obtained by summing the rating in each of six cognitive domains or 'boxes': personal care, affairs in the community and at home, judgment, orientation, and memory. Higher scores (range 0 to 18) reflect greater global impairment. To evaluate activities of daily living, we used functional rating scales: the Physical Self-Maintenance Scale (PSMS) and Instrumental Activities of Daily Living (IADL) scale [38]. These tests require input from the caregiver or an informant to evaluate the basic activities involving physical self-care (PSMS) and performance of complex daily functional activities essential to independence (IADL). The PSMS evaluates six aspects of self-care: toilet, feeding, dressing, grooming, ambulation, and bathing. Each item is scored from 1 to 5, and the maximum score is 30. The IADL evaluates eight complex tasks, including the use of the telephone, ability to shop, and mode of transportation. A score of zero can be assigned for five of the items if the patient did not perform the activity in question prior to the onset of illness: food preparation, housekeeping, laundry, ability to handle finances, and responsibility for medications. The maximum score is 31. Higher scores on the PSMS and IADL indicate greater functional impairment.

### Pre-progression rate

AD patients progress at different rates but little is known about factors that explain the variance. The pre-progression rate is an easily calculable index of early disease progression and has prognostic value in classifying patients as rapid, intermediate, or slow progressors. It is calculated as (the MMSE score [expected 30] - MMSE score [initial])/physician's estimate of symptom duration (in years). The pre-progression rate is significant in determining time to clinically meaningful decline during longitudinal follow-up [39,40]. It was therefore an important covariate in the current analysis.

### Covariates

Age, gender, years of education, baseline severity of dementia (mild, moderate, or severe based on MMSE score), pre-progression rate, and an indicator variable reflecting whether a patient had started on antidementia therapy before their initial visit to the Alzheimer's Disease and Memory Disorders Center. The use of drug prior to the initial visit (early exposure index) was used to control for the effects of early treatment or the propensity to take medication or both.

### Statistical analysis

Descriptive statistics (that is, frequencies for categorical variables and means and standard deviations for continuous variables) for the following variables were determined for the group as a whole at baseline: gender, age, education, physician's estimate of symptom duration, early exposure index, MMSE, ADAS-Cog, PSMS, IADL, CDR-SB, and BPMSE. Repeated measurement (random effects) linear modeling was used to examine the impact of cumulative antidementia drug exposure (PI) on change in the slope or rate of decline for the neuropsychological tests. Since this study focused on cohort average effect, no specific random effect was added to the model on any particular covariate, except assuming the existence of correlation between two outcome observations of the same subject. The correlation between two observations of any neuropsychological variable was assumed to follow an autoregressive correlation model of order 1. This model takes into consideration that the correlation of two observations for each outcome variable is inversely proportional to the time interval between these two measurements. The primary model included time from the first visit, PI, interaction of these two terms, and pre-progression rate at the initial visit. Adjustments were then made for age, gender, years of education, baseline severity of dementia (mild, moderate, or severe based on MMSE score), and early exposure index (categorical). The outcome data for each measure were evaluated for significance of the quadratic relationship, including both linear and quadratic trend change. The regression coefficient associated with the variable PI indicated the amount of change in cognitive/functional performance or change in mean test score associated with a unit change in the PI at baseline. The regression coefficient associated with the interaction of time from the first visit and PI (PI × time) indicated the rate of decline on neuropsychological tests or change in the slope associated with a unit change in the PI.

## Results

There were 672 eligible subjects. Among them, 31 did not have either PI or complete neuropsychological measurements at any time point and were excluded from the analysis. We compared the sample of included patients (n = 641) and those subjects who were missing an important covariate (n = 31) and found no significant differences in gender, years of education, age, or number of clinic visits (analysis not reported). The excluded subjects had a significantly longer duration of symptoms and higher early exposure index at the new patient visit (*P *< 0.01). Baseline demographic characteristics were age, gender, years of education and symptom duration, average follow-up time, number of annual visits, and the early exposure index (Table 1) and the baseline scores on the ADAS-Cog, MMSE, BPMSE, IADL, PSMS, and CDR-SB (Table 2). A frequency analysis of drug exposures in the 641 patients indicated that 43% of the group had been exposed to drug before the initial visit and 43% began treatment within 2 years following the new patient visit. The percentage of individuals who were using medication at baseline increased with the duration of symptoms. Over the entire period of follow-up, 12% never used medication (Table 3). In the longitudinal data including all visits, there were equal proportions on a ChEI alone (n = 1,623) or combination therapy of a ChEI and memantine (n = 1,627) and few taking only memantine (n = 169).

**Table 1:** Baseline characteristics of patients with Alzheimer disease

Variable (n = 641)	Value	Range
Age, years	73 (8.50)	43-93

Female	68%	

Education, years	14 (3.56)	0-29

Early exposure index, years	0.5 (0.27)	0-1

Duration of symptoms before initial visit, years	3.7 (2.29)	0.5-13

Follow-up time, years	3.0 (1.94)	0.8-13.4

Total number of visits	3.4 (1.64)	2-11

For all continuous variables, values are presented as mean (standard deviation).

**Table 2:** Baseline neuropsychological test scores

Variable (n = 641)	Average score	Range
MMSE	19.5 (6.64)	0-30

ADAS-Cog	24.3 (12.43)	1-68

BPMSE	19.6 (5.96)	0-25

CDR-SB	6.7 (4.02)	0.5-18

IADL	15.5 (6.50)	2-31

PSMS	7.9 (3.05)	6-25

For all continuous variables, values are presented as mean (standard deviation). ADAS-Cog, Alzheimer's Disease Assessment Scale-Cognitive Subscale; BPMSE, Baylor Profound Mental Status Examination; CDR-SB, Clinical Dementia Rating-Sum of Boxes; IADL, Instrumental Activities of Daily Living; MMSE, Mini-Mental State Examination; PSMS, Physical Self-Maintenance Scale.

**Table 3:** Drug exposure and duration of illness

	Treatment initiated				
**Duration of symptoms at the NPV**	**Before NPV**	**1-2 years after NPV**	**3 years after NPV**	**Never treated**	**Total**

<2 years	37 (5.8)	49 (7.6)	5 (0.8)	18 (2.8)	109 (17.0)

2-3 years	98 (15.3)	112 (17.5)	10 (1.6)	33 (5.1)	253 (39.5)

4-5 years	79 (12.3)	80 (12.5)	2 (0.3)	13 (2.0)	174 (27.1)

≥6 years	59 (9.2)	34 (5.3)	1 (0.2)	11 (1.7)	105 (16.4)

Total	273 (42.6)	275 (42.9)	18 (2.8)	75 (11.7)	641 (100.0)

Values are presented as number (percentage). NPV, new patient visit.

In a linear model, greater antidementia drug use was significantly associated with a slower rate of decline over time on the MMSE (*P *< 0.0001), ADAS-Cog (*P *< 0.01), PSMS (*P *< 0.05), IADL (*P *< 0.0001), and CDR-SB (*P *< 0.001). There was a trend (*P *= 0.053) indicating that greater antidementia drug was associated with a slower rate of decline on BPMSE (Table 4, PI × time). Assumptions of linear change were valid for all of the rating scales except the ADAS-Cog. Including both linear and quadratic terms in the model for ADAS-Cog change, we found that greater antidementia drug use was associated with a slower rate of decline on the ADAS-Cog for the first 3.3 years and that afterwards the positive treatment effect diminished.

**Table 4:** Relationship between persistency index and outcome measures (mixed effects regression analysis)

**Outcome(s) with adjustment**^a^	Intercept	Time, years	Persistency index	Persistency index × time
	**Beta coefficient^b ^(standard error)**			

MMSE	3.89 (2.001)	-2.58 (0.13)^c^	-1.09 (0.77)	1.02 (0.23)^c^

ADAS-Cog^d^	55.45 (5.65)^c^	3.68 (0.66)^c^	-3.75 (2.09)	2.74 (1.32)^e^

BPMSE	10.46 (3.47)^e^	-2.55 (0.25)^c^	-1.76 (1.90)	1.00 (0.52)

PSMS	10.03 (1.85)^c^	1.68 (0.12)^c^	-0.09 (0.66)	-0.43 (0.21)^f^

IADL	18.63 (2.54)^c^	2.36 (0.17)^c^	4.19 (0.91)^c^	-1.42 (0.29)^c^

CDR-SB	11.43 (1.43)^c^	1.67 (0.09)^c^	1.42 (0.54)^e^	-0.61 (0.17)^g^

^a^Adjustment made for early exposure index, gender, education, age, pre-progression rate, and the severity of disease at baseline. ^b^Mean change in score associated with each variable. ^c^*P *< 0.0001. ^d^When the linear trend and quadratic trend change were included in the model the coefficients (standard errors) for time-squared and time-squared by persistency index interaction are 0.19 (0.14) and -0.83 (0.26)^e^, respectively. ^e^*P *< 0.01; ^f^*P *< 0.05; ^g^*P *< 0.001; otherwise, *P *= not significant, based on type 3 F test. ADAS-Cog, Alzheimer's Disease Assessment Scale-Cognitive Subscale; BPMSE, Baylor Profound Mental Status Examination; CDR-SB, Clinical Dementia Rating-Sum of Boxes; IADL, Instrumental Activities of Daily Living; MMSE, Mini-Mental State Examination; PSMS, Physical Self-Maintenance Scale.

The magnitude of the treatment effect was clinically relevant. The rate of change in mean test scores indicated that maximally treated compared with untreated patients would have less decline on the rating scales: 1 point per year on the MMSE, 0.4 points per year on the PSMS, 1.4 points per year on the IADL, and 0.6 points per year on the CDR-SB. These treatment effects are additive over the entire period of observation. After 5 years, maximally treated patients would retain 4 more points on the MMSE, 2 fewer points on the PSMS, 3 fewer points on the IADL, and 1.6 fewer points on the CDR-SB. The benefits are also reflected in the ADAS-Cog, but properties of this test suggest that after about 3 years the persistent treatment effect is no longer detectable. Overall, there appears to be incremental benefit associated with greater cumulative antidementia drug use.

## Discussion

Patients who received more persistent exposure to the antidementia drugs over the course of their illness had a significantly slower rate of decline on key measures of cognition, global functioning, and basic activities of daily living. These effects are cumulative over time. Our results are consistent with a recent 3-year prospective study that compared outpatients taking ChEIs with untreated historical controls and that found that treatment was associated with fewer declines on a global dementia rating scale (Clinician Interview Based Impression of Change) and with significantly improved cognition as assessed by the MMSE and ADAS-Cog [41]. Our study indicates that persistent treatment is associated with positive benefit over the entire course of the disease and is reflected in both cognitive and functional outcomes.

One shortcoming of this study is that drug exposure was not assigned randomly, so we could not explore potential differences in combinations of antidementia drugs. In addition, we did not have data on indications for treatment or MMSE scores before the patients were first evaluated at our center. The PI was constant (not time-dependent) and did not reflect the outcome for each patient at each time point. PI as determined by the last observation was related in the analysis to earlier cognitive assessments. However, our objective was to evaluate the cumulative benefit of therapy over the life span rather than short-term treatment effects. Uncontrolled variables related to disease severity or duration of symptoms or both, perceived benefits from treatment, or other unmeasured factors (including behavioral symptoms or medical comorbidity) may have influenced the observed relationship between persistency of drug use and outcomes. The fact that our study included both treated and untreated individuals, even in subcohorts examined by duration of symptoms and total follow-up time (Table 3), contributes to the validity of the findings.

It is possible that individuals who take the antidementia drugs consistently have naturally slow disease progression and that their milder disease course could be falsely attributed to drug treatment. We cannot rule out the possibility that selection factors related to disease severity or perceived benefits from treatment influenced the observed relationship between persistency of drug use and outcomes. We adjusted the analysis by calculating an early exposure index to correct for the propensity to take medication, but this adjustment may not fully control the confounding factor. In an exploratory model that adjusted for the duration of symptoms rather than the pre-progression rate, we found that taking drug before the first visit was associated with worse cognitive and functional tests yet that cumulative drug use remained beneficial. This suggests that more rapid progressors, rather than slow progressors, are more likely to have been started on drug prior to the initial visit.

## Conclusions

This study suggests that the more persistent that patients are and the longer that they persist with treatment the better they will perform on cognitive, global, and functional outcomes. The modest findings of one long-duration trial (AD2000) that included regular washouts of treatment might be explained, in part, by lack of treatment persistence [16]. The impact of AD therapy should be judged by the cumulative benefit over the duration of the disease. Our findings also suggest that antidementia drugs may benefit patients even when given in advanced or profound stages of AD.

## Abbreviations

AD: Alzheimer disease; ADAS-Cog: Alzheimer's Disease Assessment Scale-Cognitive Subscale; BCM: Baylor College of Medicine; BPMSE: Baylor Profound Mental Status Examination; CDR-SB: Clinical Dementia Rating-Sum of Boxes; ChEI: cholinesterase inhibitor; IADL: Instrumental Activities of Daily Living; MMSE: Mini-Mental State Examination; PI: persistency index; PSMS: Physical Self-Maintenance Scale.

## Competing interests

SDR has been the principal investigator of a grant award to her institution from the Forest Research Institute (Jersey City, NJ, USA), is the principal investigator of clinical trial grants from Medivation (San Francisco, CA, USA) and Sonexa Therapeutics (San Diego, CA, USA), and has been a consultant to Eisai (Woodcliff Lake, NJ, USA), Forest Laboratories, Inc. (New York, NY, USA), Novartis (Basel, Switzerland), and Pfizer Inc (New York, NY, USA). RSD has been the principal investigator for clinical trials granted to her institution from Eisai, is the principal investigator of clinical trial grants from Pfizer Inc, Elan Corporation (Dublin, Ireland), and Wyeth (Madison, NJ, USA), and has been a consultant to Eisai, Forest Research Institute, Novartis, and Pfizer Inc. The other authors declare that they have no competing interests.

## Authors' contributions

SDR conceptualized and designed the study, drafted the manuscript, and has given final approval of the version to be published. WC, VNP, and RSD made substantial contributions to the conception and design of the study and to the analysis and interpretation of data. EJD and SS assisted with the acquisition of data and the analysis. All authors read and approved the final manuscript.
